# A new, widely distributed species of the *Exocelina ekari*-group from West Papua (Coleoptera, Dytiscidae, Copelatinae)

**DOI:** 10.3897/zookeys.554.6065

**Published:** 2016-01-18

**Authors:** Helena Shaverdo, Rawati Panjaitan, Michael Balke

**Affiliations:** 1 Naturhistorisches Museum, Burgring 7, A-1010 Vienna, Austria; 2 Department of Biology, Faculty of Sciences and Mathematics, State University of Papua (UNIPA), Jalan Gunung Salju Amban, Manokwari 98314, West Papua, Indonesia; 3 Zoologische Staatssammlung München, Münchhausenstraße 21, D-81247 Munich, Germany and GeoBioCenter, Ludwig-Maximilians-University, Munich, Germany

**Keywords:** Coleoptera, Dytiscidae, Exocelina, new species, new subspecies, molecular phylogenetics, West Papua

## Abstract

*Exocelina manokwariensis* **sp. n.** from West Papua is placed into the *Exocelina ekari*-group based on the structure of its male genitalia. The new species is described, including its three subspecies, from the mainland of West Papua, Waigeo Island, Batanta and Salawati Islands, and Bomberai peninsula. An identification key to the subspecies as well as data on species distribution are provided.

## Introduction

Of the 88 species of the genus *Exocelina* Broun, 1886 described from New Guinea, only eight species are known from West Papua, and all of them belong to the *Exocelina ekari*-group (Balke 1998, 1999, Shaverdo and Balke 2014, Shaverdo et al. 2005, 2012, 2013, 2014). Herein, a new member of that group is described, which is widely distributed across the Bird's Head of West Papua, accounting for the geographical structure observed in the morphological characters studied by erecting four subspecies.

## Material and methods

The present work is based on the material from the following collections:

MZB
Museum Zoologicum Bogoriense, Cibinong, Indonesia 


NHMW
Naturhistorisches Museum Wien, Vienna, Austria 


ZSM
 Zoologische Staatsammlung München, Munich, Germany 


All specimen data are quoted as they appear on the labels attached to the specimens. Label text is cited using quotation marks. Comments in square brackets are ours. All types of the herein described specimens are provided with red labels. Female specimens, identification of which is difficult or sometimes impossible, were included in the type series only when they were collected with males of respective species and did not show external morphological differences from them. If two or more morphologically similar species were collected together (i.e., males found together), their females were not included in the type series but were instead mentioned under additional material. Species descriptions are based on the whole type series.

Measurements were taken with a Wild M10 stereomicroscope. The following abbreviations were used: TL (total body length), TL-H (total body length without head), MW (maximum body width), and hw (handwritten). Number of the ventral setae of the male protarsomere 5 is given only for one specimen of each species, which was mounted on a glass slide (see below) for drawing. This character was found to be of limited practical use for species identification since it is possible to make a general statement on the setation pattern (short/long, dense/sparse) but not to count them with certainty at the magnification of normal dissecting microscopes. The potential phylogenetic information content of this character will be studied in a further work.

Drawings were made with the aid of a camera lucida attached to a Leica DM 2500 microscope. For detailed study and drawing, antennae, protarsi, and genitalia were removed and mounted on glass slides with DMHF (dimethyl hydantoin formaldehyde) as temporary preparations. The drawings were scanned and edited, using the software Adobe Illustrator CS5.1.

The terminology to denote the orientation of the genitalia (ventral for median lobe and dorsal and external for paramere) follows Miller and Nilsson (2003). The terminology on the structure of the prosternum follows Larson et al. (2000). Administrative divisions of Indonesia follow information from Wikipedia (2015).

## Systematics

### Exocelina manokwariensis sp. n.

Taxon classificationAnimaliaColeopteraDytiscidae

http://zoobank.org/77D1CC24-24BC-4BE1-AC1F-CBAEA851B583

Figs 1 , 2

#### Type locality.

Indonesia: West Papua Province: Manokwari Regency, Road Manokwari – Mokwam, 01°00.60'S; 133°53.92'E.

**Type material.** *Holotype*: male “Indonesia: Papua, Road Manokwari - Mokwam, 320m, 25./27.i.1990, 01.00.596S 133.53.921E, Balke (BH 01)” (MZB). *Paratypes*: **Manokwari**: 12 males, 8 females with the same label as the holotype, two males additionally with green labels “M.Balke 1267”, “M.Balke 1282” (MZB, NHMW, ZSM). 6 males, 5 females “Indonesia: Papua, Ransiki - Anggi, 1160m, 30.i.1990, 01.25.536S 134.02.456E, Balke (BH 03)”, one male additionally with a green label “M.Balke 1270” (NHMW, ZSM). 2 males, 5 females “IRIAN JAYA, Manokwari Rasiki, Mayuby – Benyas ca. 300–400m, 27.-28.IX.1990, leg. A. Riedel” (NHMW, ZSM). 121 males, 94 females “Indonesia: Papua Barat, Manokwari, Maripi, creek white pebbles, 135m, -0.907576 133.9214718 (BH039)” (MZB, NHMW, ZSM). 2 males “IN: West Papua: Manokwari Reg., on road Manokwari-Kebar, near Munbrani vill., 66 m, 8.V., 00°46'21"S, 133°22'53"E,, roadside ditch (2015-WP36)” (NHMW, ZSM). 1 male “Indonesia: Papua Barat, Manokwari to Kebar, forest stream, 302m, -0.80058566 133.33216397 (BH023)”, one male additionally with a label “M.Balke 6187” (ZSM). 36 males “Indonesia: Papua Barat, Kebar to Aibogar, slow forest stream, 503m, -0,86241595 132,82993928 (BH025)”, one male additionally with a label “M.Balke 6190” (MZB, NHMW, ZSM). 2 males, 4 females “Indonesia: Papua Barat, Kebar to Aibogar, forest stream, 942m, -0,89933965 132,7221734 (BH026)”, one male additionally with a label “M.Balke 6192” (ZSM). 38 males, 26 females “Indonesia: Papua Barat, Fumato, forest stream, 820m, -0.90427148 132.71981431 (BH027)”, four males additionally with labels “M.Balke 6201”, “M.Balke 6202”, “M.Balke 6203”, “M.Balke 6204” (MZB, NHMW, ZSM). 31 males, 22 females “Indonesia: Papua Barat, Fumato to Kebar, forest stream, 674m, -0.88384738 132.73705681 (BH028)” (MZB, NHMW, ZSM). 11 males, 7 females “Indonesia: Papua Barat, Tamrau Mts. N of Kebar, forest stream, 750m, -0,783199 133,072143 (BH033)” (ZSM). 75 males, 154 females “Indonesia: Papua Barat, Tamrau Mts. N of Kebar, forest stream, puddles, 1050m, -0.774519 133.069929 (BH034)” (MZB, NHMW, ZSM). **Sorong**: 6 males, 9 females “Indonesia: Papua Barat, Sorong-Sausapor, 300m, -0.7629653 131.6177023 (BH041)” (ZSM). 18 males, 21 females “Indonesia: Papua Barat, Sausapor-Fef, 157m, -0.6975004 132.072253 (BH044)” (MZB, NHMW, ZSM).

#### Additional material.

1 female “Indonesia: Papua Barat, Tamrau Mts. N of Kebar, sandy sunny riverbank”, “758m, -0,78387424 133,07218533 (BH032)” (ZSM). 78 females “Indonesia: Papua Barat, Kebar to Aibogar, slow forest stream, 503m, -0,86241595 132,82993928 (BH025)”, the females are a mixture of *Exocelina manokwariensis* sp. n. and *Exocelina polita* (Sharp, 1882) (MZB, NHMW, ZSM).

#### Diagnosis.

Beetle small, brown to blackish brown, usually with paler clypeus and pronotal sides or head and pronotum, shiny, with almost invisible dorsal punctation; pronotum with distinct lateral bead; male antennomeres 3–4 strongly enlarged and triangular (3 distinctly larger than 4), 5 distinctly enlarged, 6–8 somewhat enlarged; male protarsomere 4 with medium-sized, slender, evidently curved anterolateral hook-like seta; median lobe with strong submedian constriction in ventral view, apex of median lobe truncate; paramere with notch on dorsal side and subdistal part short and small, with relatively short, thick, and flattened setae.

#### Description.

*Size and shape*: Beetle small (TL-H 3.2–3.75 mm, TL 3.45–4.15 mm, MW 1.7–2.05 mm; *holotype*: TL-H 3.3 mm, TL 3.7 mm, MW 1.75 mm), with oblong-oval habitus, broadest at elytral middle.

**Figure 1.**
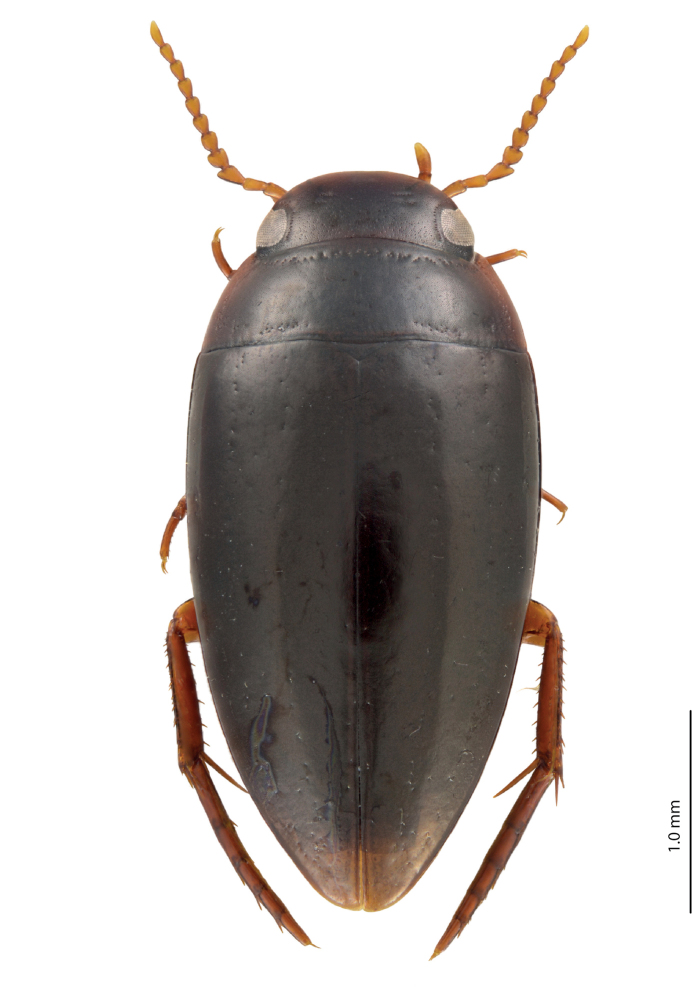
Habitus and coloration of *Exocelina manokwariensis* sp. n.

*Coloration*: Head reddish brown to blackish brown, paler on clypeus and vertex; pronotum reddish brown to blackish brown, with paler sides and darker disc; elytra brown to blackish brown, sometimes with brown sutural lines; head appendages and legs yellowish to yellowish red, legs darker distally (Fig. 1). Teneral specimens paler.

*Surface sculpture*: Head with relatively dense punctation (spaces between punctures 1–3 times size of punctures), evidently finer and sparser anteriorly; diameter of punctures smaller than diameter of cells of microreticulation. Pronotum with extremely sparse and fine punctation, almost invisible. Elytra with punctation finer than on pronotum, indistinct. Pronotum and elytra with weakly impressed microreticulation, dorsal surface shiny. Head with microreticulation stronger. Metaventrite and metacoxa distinctly microreticulate, metacoxal plates with longitudinal strioles and transverse wrinkles. Abdominal ventrites with distinct microreticulation, strioles, and extremely fine, sparse punctation, almost invisible, only slightly coarser and denser on two last abdominal ventrites.

*Structures*: Pronotum with distinct lateral bead. Base of prosternum and neck of prosternal process with distinct ridge, less rounded and smooth anteriorly, without anterolateral extensions. Blade of prosternal process lanceolate, relatively elongate, convex, with distinct lateral bead and few setae; neck and blade of prosternal process evenly jointed. Abdominal ventrite 6 broadly rounded or slightly truncate apically.

**Figure 2.**
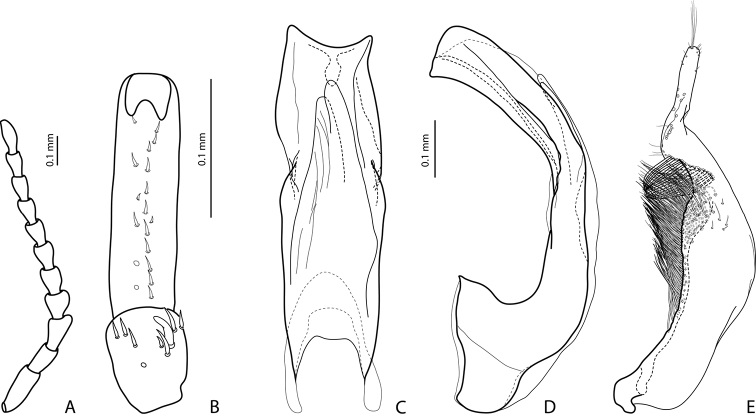
*Exocelina manokwariensis* sp. n. **A** male antenna **B** male protarsomeres 4–5 in ventral view **C** median lobe in ventral view **D** median lobe in lateral view **E** paramere in external view.

*Male*: Antennomeres 3–4 strongly enlarged and triangular (3 distinctly larger than 4), 5 distinctly enlarged, 6–8 somewhat enlarged (Fig. 2A); antennomeres 3–7 rugose ventrally. Protarsomere 4 with medium-sized, slender, evidently curved anterolateral hook-like seta. Protarsomere 5 ventrally with anterior row of 12 and posterior row 6 short setae (Fig. 2B). Abdominal ventrite 6 with 8–16 lateral striae on each side, slightly truncate apically. Median lobe with strong submedian constriction in ventral view, apex of median lobe truncate, with relatively short tip in lateral view and relatively asymmetric in ventral view (Fig. 2C, D). Paramere with notch on dorsal side and subdistal part short and small, with numerous, relatively short, thick, and flattened setae (Fig. 2E).

*Female*: Antennae simple, abdominal ventrite 6 broadly rounded apically, without striae.

#### Variability.

The species has three subspecies, which are isolated geographically, occurring in restricted regions (Fig. 9). They are very similar morphologically and show (at least on material we have) no much variability in their morphology. Comparisons are given as separate notes after the descriptions.

Here, we discuss the variability of the nominative subspecies, which is more widely distributed (Fig. 9). This subspecies demonstrates variability mainly in shape of the apex of median lobe and setation of the subdistal part of the paramere. The truncate margin of the median lobe apex varies from almost straight (especially specimens from Manokwari) to slightly concave (especially specimens from Kedar, Fumato, Sorong) in lateral view. This variability is observed within and among the populations. Also, number of the subdistal flattened setae of the paramere varies thought it is not possible to estimate that quantitatively, since they are numerous and densely attached. The specimens from western populations have less numerous subdistal flattened setae then specimens from Manokwari.

#### Comparative notes.

In the *Exocelina ekari*-group, the new species is similar to the species of the *Exocelina polita*-complex: *Exocelina polita* (Sharp, 1882), *Exocelina alexanderi* Shaverdo, Hendrich & Balke, 2012, *Exocelina anggiensis* Shaverdo, Hendrich & Balke, 2012, and *Exocelina arfakensis* Shaverdo, Hendrich & Balke, 2012; see descriptions and illustrations in Shaverdo et al. 2012. From all these species, *Exocelina manokwariensis* sp. n. can be distinguished by its smaller size (TL-H: 3.1–3.85 mm, MW: 1.65–2.1 mm; for *Exocelina polita*-complex: TL-H: 3.7–4.3 mm, MW: 2.05–2.3 mm) and apex of the median lobe almost truncate in lateral view (elongate for *Exocelina polita*-complex, if slightly truncate (in *Exocelina alexanderi*), then anntenomeres 3 and 4 of almost equal size). With its very fine dorsal punctation, the new species particularly resembles *Exocelina alexanderi*, which also has a slightly truncate apex of the median lobe in lateral view, but differs from it by the smaller size and distinctly different shape of the male antennomeres 3 and 4. *Exocelina polita* also has fine dorsal punctation and it was found together with the new species in two localities. From *Exocelina polita*, *Exocelina manokwariensis* sp. n. can be separated by its smaller size, truncate apex of the median lobe in lateral view, and slightly different shape of the male antennomeres 3 and 4.

The nominative subspecies can be distinguished from all the other subspecies by more numerous flattened setae on the subdistal part of the paramere, the slightly shorter median lobe, and the prosternal ridge being less rounded anteriorly. From *Exocelina manokwariensis batanta* ssp. n. and *Exocelina manokwariensis nokensis* ssp. n., it can be also separated by slightly darker coloration and the stronger dorsal microreticulation. From *Exocelina manokwariensis batanta* ssp. n. and *Exocelina manokwariensis hendrichi* ssp. n., by the more asymmetric apex of the median lobe in ventral view and the smaller subdistal part of the paramere.

#### Distribution.

Indonesia: West Papua Province: Sorong and Manokwari Regencies (Fig. 9).

#### Etymology.

The species is named after Manokwari Regency where it is occur. The name is an adjective in the nominative singular.

#### Ecology.

The species was collected in localities BH 023 and BH 025 together with *Exocelina polita* in ratios 1:4 and 1:1, respectively.

### Exocelina manokwariensis batanta ssp. n.

Taxon classificationAnimaliaColeopteraDytiscidae

http://zoobank.org/047A24B7-0ED0-491E-BF10-B69C103B2236

Figs 3 , 4

Exocelina undescribed sp. MB1277: Toussaint et al. 2014: Supplementary figs 1–4, tab. 2.

#### Type locality.

Indonesia: West Papua Province: Raja Ampat Regency, Batana Utara, approximately 00°50.13'S; 130°42.86'E.

#### Type material.

*Holotype*: male “Indonesia: Papua, Batanta Utara, 180m, above 00.50.125S 130.42.856E (BH 13)” (MZB). *Paratype*: 16 males, 14 females with the same label as the holotype, two males additionally with green labels “M.Balke 1277”, “M.Balke 1278” (MZB, NHMW, ZSM). 3 males, 4 females “Indonesia: Papua, Batanta Selatan, Wailebet, 280m, inland 00.53.957S 130.39.951E, (BH 16)”, one male additionally with a green label “M.Balke 1280” (NHMW, ZSM). 2 males, 2 females “Indonesia: Papua, Salawatti Utara, 100-250m, inland 00.57.954S 130.40.531E (BH 18)”, one male additionally with a green label “M.Balke 1281” (NHMW, ZSM).

#### Description.

As nominative subspecies, except for the following characters.

*Size*: TL-H 3.2–3.6 mm, TL 3.65–3.95 mm, MW 1.75–1.9 mm; *holotype*: TL-H 3.5 mm, TL 3.85 mm, MW 1.85 mm.

**Figure 3.**
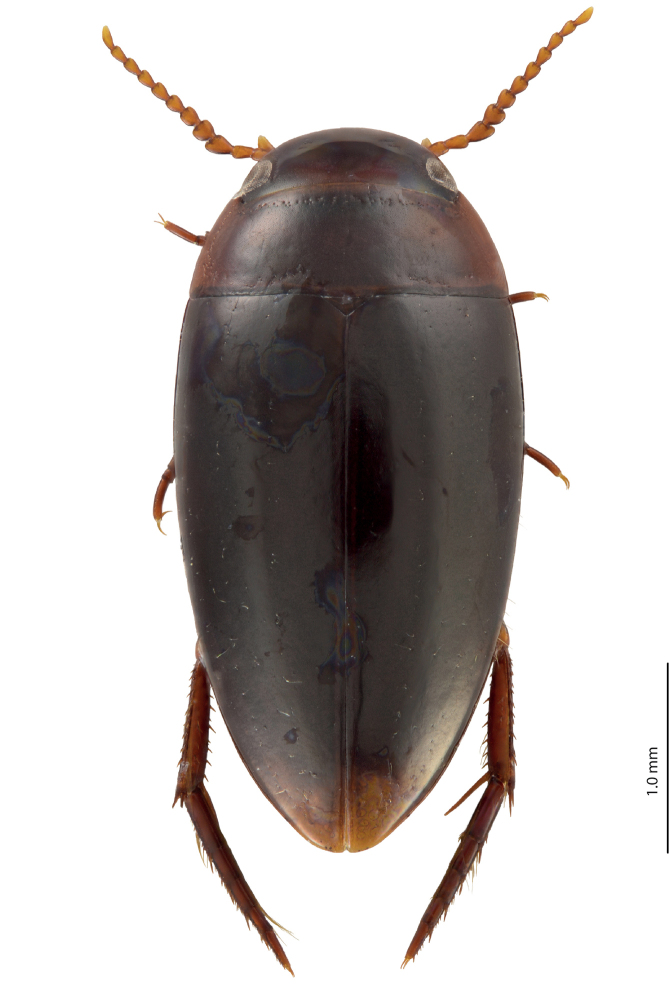
Habitus and coloration of *Exocelina manokwariensis batanta* ssp. n.

*Coloration*: Head reddish brown to dark brown, paler on clypeus and vertex; pronotum reddish brown to dark brown, with paler sides and darker disc; elytra brown to blackish brown, usually with reddish brown sutural lines (Fig. 3).

*Surface sculpture*: Dorsal microreticulation, especially on head and pronotum, slightly weaker.

*Structures*: Base of prosternum and neck of prosternal process with distinct ridge, rounded and smooth anteriorly, with few transverse lines.

**Figure 4.**
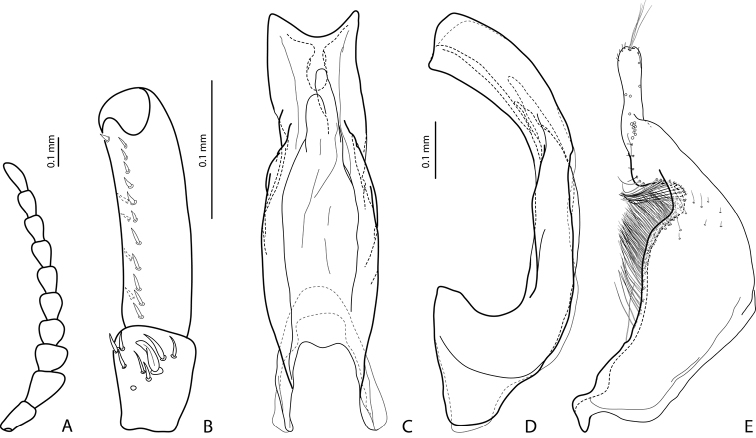
*Exocelina manokwariensis batanta* ssp. n. **A** male antenna (antennomere 1 not shown) **B** male protarsomeres 4–5 in ventral view **C** median lobe in ventral view **D** median lobe in lateral view **E** paramere in external view.

*Male*: Antennomeres 3–4 strongly enlarged and triangular (3 distinctly larger than 4), 5–6 distinctly enlarged, 7–8 somewhat enlarged (Fig. A). Protarsomere 5 ventrally with anterior row of 11 and posterior row 5 short setae (Fig. 4B). Abdominal ventrite 6 with 6–8 lateral striae on each side. Median lobe larger. Its apex strongly concave and symmetric in ventral view and with truncate margin more strongly curved in lateral view (Fig. 4C, D). Subdistal part of paramere larger, with flattened setae thinner and less numerous (Fig. 4E).

*Female*: Antennae simple, abdominal ventrite 6 broadly rounded apically, without striae.

#### Comparation with the other subspecies.

*Exocelina manokwariensis batanta* ssp. n. can be separated from all other subspecies by its less numerous and thinner flattened setae of the subdistal part of the paramere and the apex of the median lobe being strongly concave and symmetric in ventral view.

#### Distribution.

Indonesia: West Papua Province: Raja Ampat Regency, Batanta Island and the northern part of Salawati Island (misspelled as Salawatti on labels) (Fig. 9). *Exocelina manokwariensis batanta* ssp. n. is the only known *Exocelina* species in these islands.

#### Etymology.

The species is named for the Batanta Island, where it was discovered. The name is a noun in the nominative singular standing in apposition.

### Exocelina manokwariensis hendrichi ssp. n.

Taxon classificationAnimaliaColeopteraDytiscidae

http://zoobank.org/4EC8EF55-8ED9-40CC-A6C4-DBC1CDA45F8E

Figs 5 , 6

Exocelina undescribed sp. MB1321: Toussaint et al. 2014: Supplementary figs 1–4, tab. 2.

#### Type locality.

Indonesia: West Papua Province: Fak-Fak Regency, Kalimati, 4 km N Fak-Fak, approximately 02°53.76'S; 132°18.07'E.

#### Type material.

*Holotype*: male “IRIAN JAYA: Fak-Fak Kalimati, 4 km N Fak-Fak 260 m, 8–9.8.1991 Balke & Hendrich (IR 27)” (MZB). *Paratype*: 26 males, 22 females with the same label as the holotype, one female additionally with a green label “M.Balke 3261” (MZB, NHMW, ZSM). 6 males “IR 27-W.New Guinea, Fak-Fak, Kali Mati 4km N F.-F., 260 m, 8.-9.viii.1991 Balke & Hendrich leg.” ((NHMW, ZSM). 44 males, 31 females “West New Guinea/Fak-Fak/IR27 Kali Mati, 4 km N of Fak-Fak 260 m, 8. & 9.8.1991 leg: Balke & Hendrich” (NHMW, ZSM). 2 males, 1 female “Indonesia: Irian Jaya Barat, Fak Fak, 310m, 23.ii.2006, Tindige”, with green labels “M.Balke 1321”, “M.Balke 4190”, “M.Balke 4191” respectively (ZSM). 1 female “Indonesia: Irian Jaya Barat, Fak Fak, 310m, 23.ii.2006, 2 53.756S 132 18.07E, Tindige (Fak-Fak)” (ZSM).

#### Description.

As nominative subspecies, except for the following characters.

*Size*: TL-H 3.3–3.85 mm, TL 3.75–4.45 mm, MW 1.75–2.1 mm; *holotype*: TL-H 3.75 mm, TL 4.3 mm, MW 2 mm.

**Figure 5.**
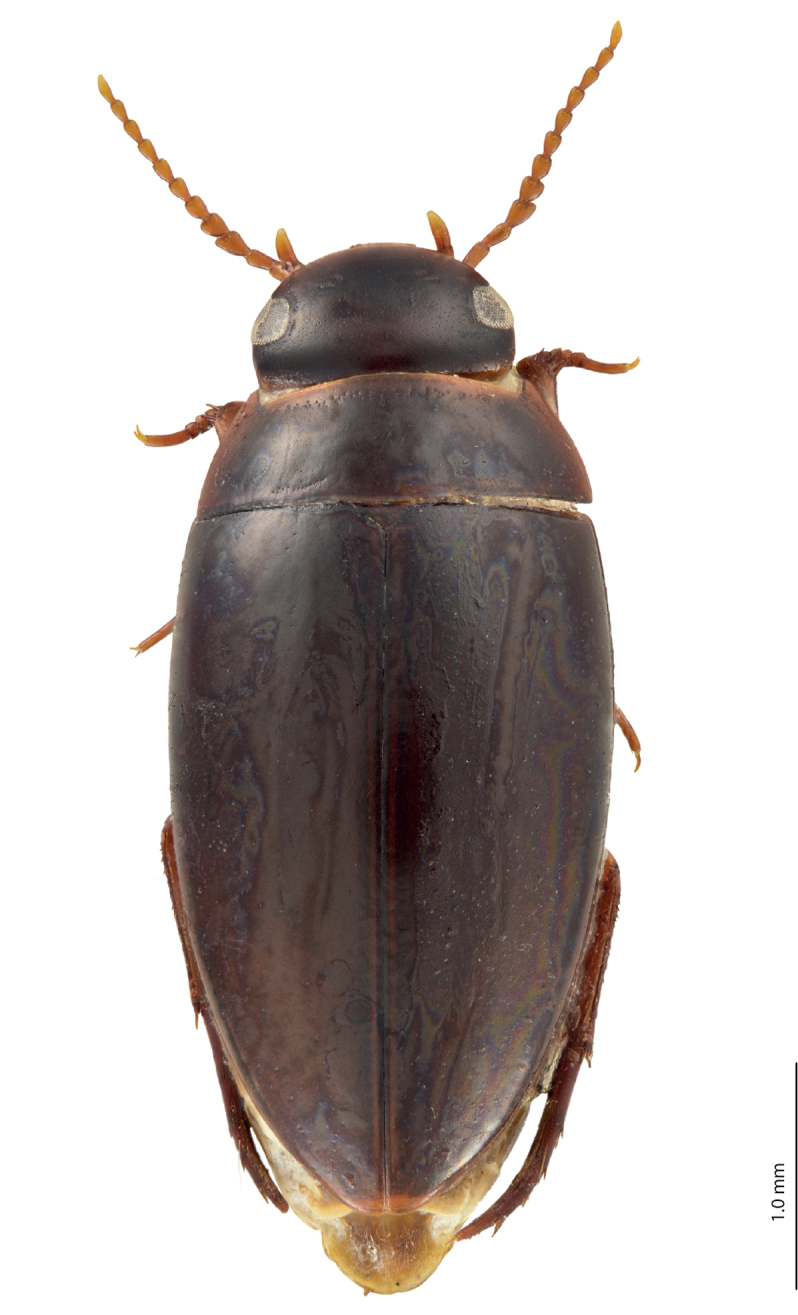
Habitus and coloration of *Exocelina manokwariensis hendrichi* ssp. n.

*Coloration*: Dorsal surface more or less uniform reddish brown to dark brown, paler on clypeus, pronotal sides, along elytral suture, and sometimes on vertex (Fig. 5).

*Structures*: Base of prosternum and neck of prosternal process with distinct ridge, rounded and smooth anteriorly.

**Figure 6.**
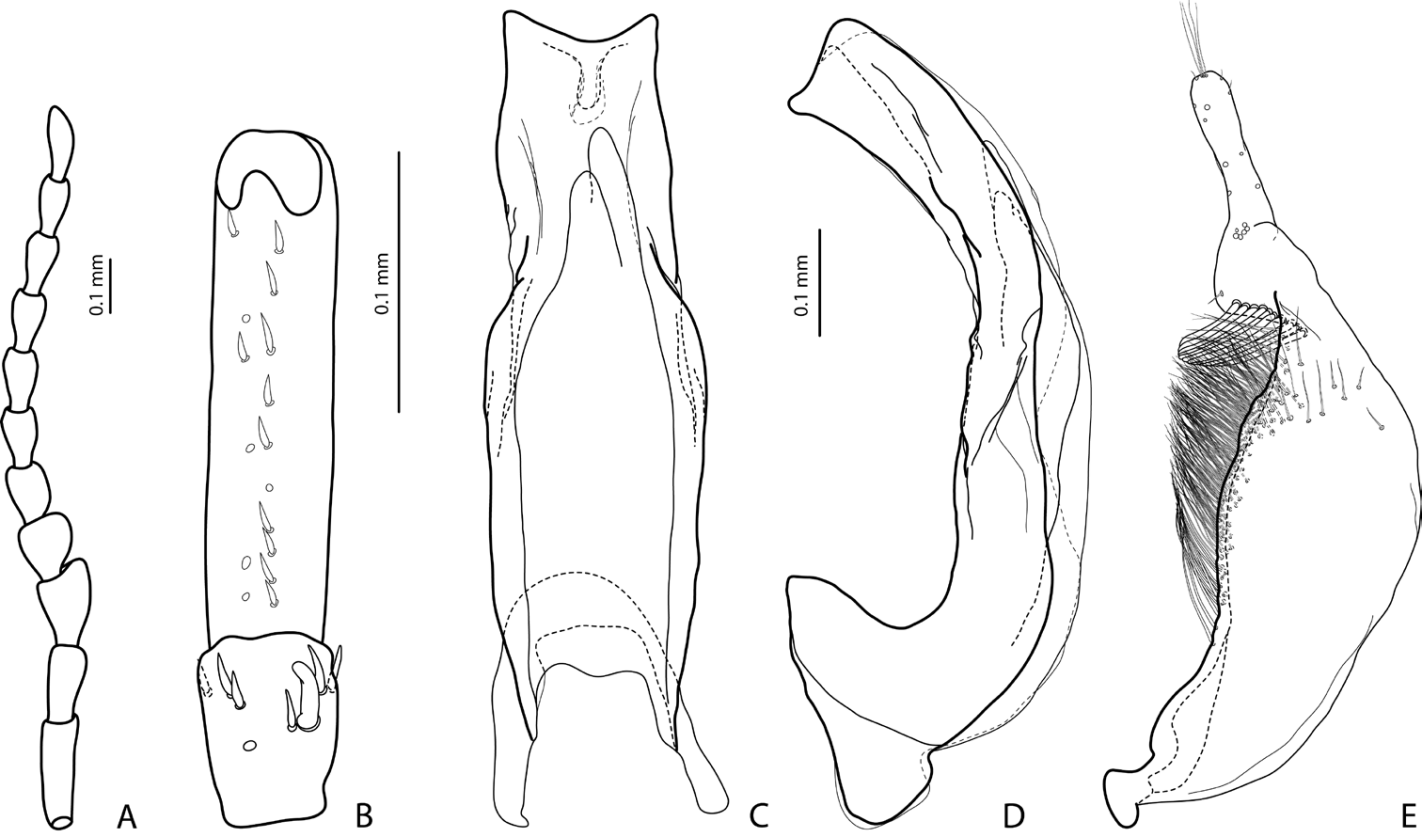
*Exocelina manokwariensis hendrichi* ssp. n. **A** male antenna **B** male protarsomeres 4–5 in ventral view **C** median lobe in ventral view **D** median lobe in lateral view **E** paramere in external view.

*Male*: Antennomeres 3–4 strongly enlarged and triangular (3 distinctly larger than 4), 5 distinctly enlarged, 6–7 somewhat enlarged (Fig. 6A). Protarsomere 5 ventrally with anterior row of 10 and posterior row 6 short setae (Fig. 6B). Abdominal ventrite 6 with 7–9 lateral striae on each side, slightly truncate apically. Median lobe larger. Its apex slightly asymmetric in ventral view and concave, with relatively long tip in lateral view (Fig. 6C, D). Subdistal part of paramere larger, with more or less numerous, thick flattened setae (Fig. 6E).

*Female*: Antennae simple, abdominal ventrite 6 broadly rounded apically, without striae.

#### Comparation with the other subspecies.

*Exocelina manokwariensis hendrichi* ssp. n. can be separated from all other subspecies by the shape of the apex of its median lobe: slightly asymmetric in ventral view and concave, with relatively long tip in lateral view.

#### Distribution.

Indonesia: West Papua Province: Fak-Fak Regency (Fig. 9). *Exocelina manokwariensis hendrichi* ssp. n. is the only known *Exocelina* species in this region.

#### Etymology.

The species is named for our colleague and friend Lars Hendrich who collected this species. The species name is a noun in the genitive case.

### Exocelina manokwariensis nokensis ssp. n.

Taxon classificationAnimaliaColeopteraDytiscidae

http://zoobank.org/9F65FAC4-58E8-4667-9C12-236CB7F46016

Figs 7 , 8

Exocelina undescribed sp. MB1275: Toussaint et al. 2014: Supplementary figs 1–4, tab. 2.

#### Type locality.

Indonesia: West Papua Province: Raja Ampat Regency, Waigeo Island, Waifoi, Mountain Nok, 00°05.08'S; 130°44.59'E.

#### Type material.

*Holotype*: male “Indonesia: Papua, Waigeo, Waifoi, Mt. Nok, 500m, 00.05.076S 130.44.586E (BH 11)” (MZB). *Paratypes*: 25 males, 39 females with the same labels as the holotype, one male additionally with a green label “M.Balke 1275” (MZB, NHMW, ZSM). 1 male “Indonesia: Papua, Waigeo, Waifoi, <50m, 00.06.088S 130.42.855E, Balke (BH 10)”, “M.Balke 1274” [green] (ZSM). 23 males “N.DUTCH NEW GUINEA: Waigeu. Camp Nok. 2,500 ft. iv.1938. L.E.Cheesman. B.M.1938-593.”, one of them additionally with labels “collection 28”, “measured J.Parkin 78” (BMNH). 5 males “N.DUTCH NEW GUINEA: Waigeu.Camp 1.Mt.Nok. 2,500 ft. v.1938. L.E.Cheesman. B.M.1938-593.” (BMNH).

#### Additional material.

27 females “N.DUTCH NEW GUINEA: Waigeu. Camp Nok. 2,500 ft. iv.1938. L.E.Cheesman. B.M.1938-593.”, one of them additionally with labels “collection 27”, “measured J.Parkin 76” (BMNH). 6 females “N.DUTCH NEW GUINEA: Waigeu.Camp 1.Mt.Nok. 2,500 ft. v.1938. L.E.Cheesman. B.M.1938-593.” (BMNH). These females might belong to two species: *Exocelina manokwariensis nokensis* ssp. n. and *Exocelina waigeoensis* Shaverdo, Hendrich & Balke, 2012.

#### Description.

As nominative subspecies, except for the following characters.

**Figure 7.**
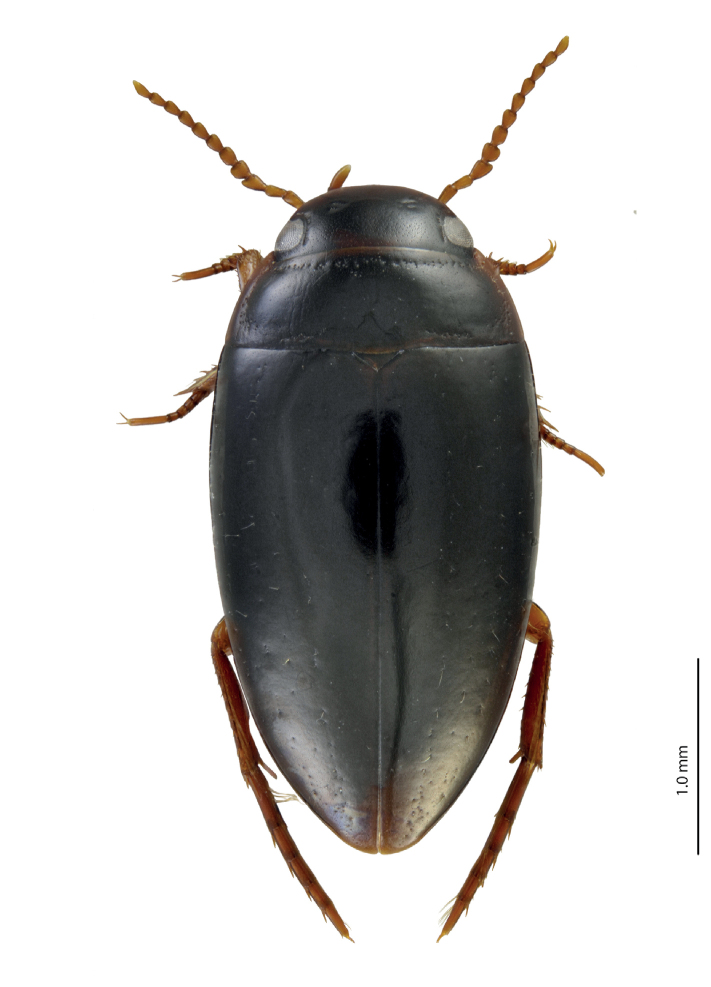
Habitus and coloration of *Exocelina manokwariensis nokensis* ssp. n.

*Size*: TL-H 3.1–3.6 mm, TL 3.45–4.0 mm, MW 1.65–1.9 mm; *holotype*: TL-H 3.4 mm, TL 3.75 mm, MW 1.8 mm (Fig. 7).

*Surface sculpture*: Dorsal microreticulation, especially on head and pronotum, slightly weaker.

*Structures*: Base of prosternum and neck of prosternal process with distinct ridge, less rounded, smooth or with few transverse lines anteriorly.

**Figure 8.**
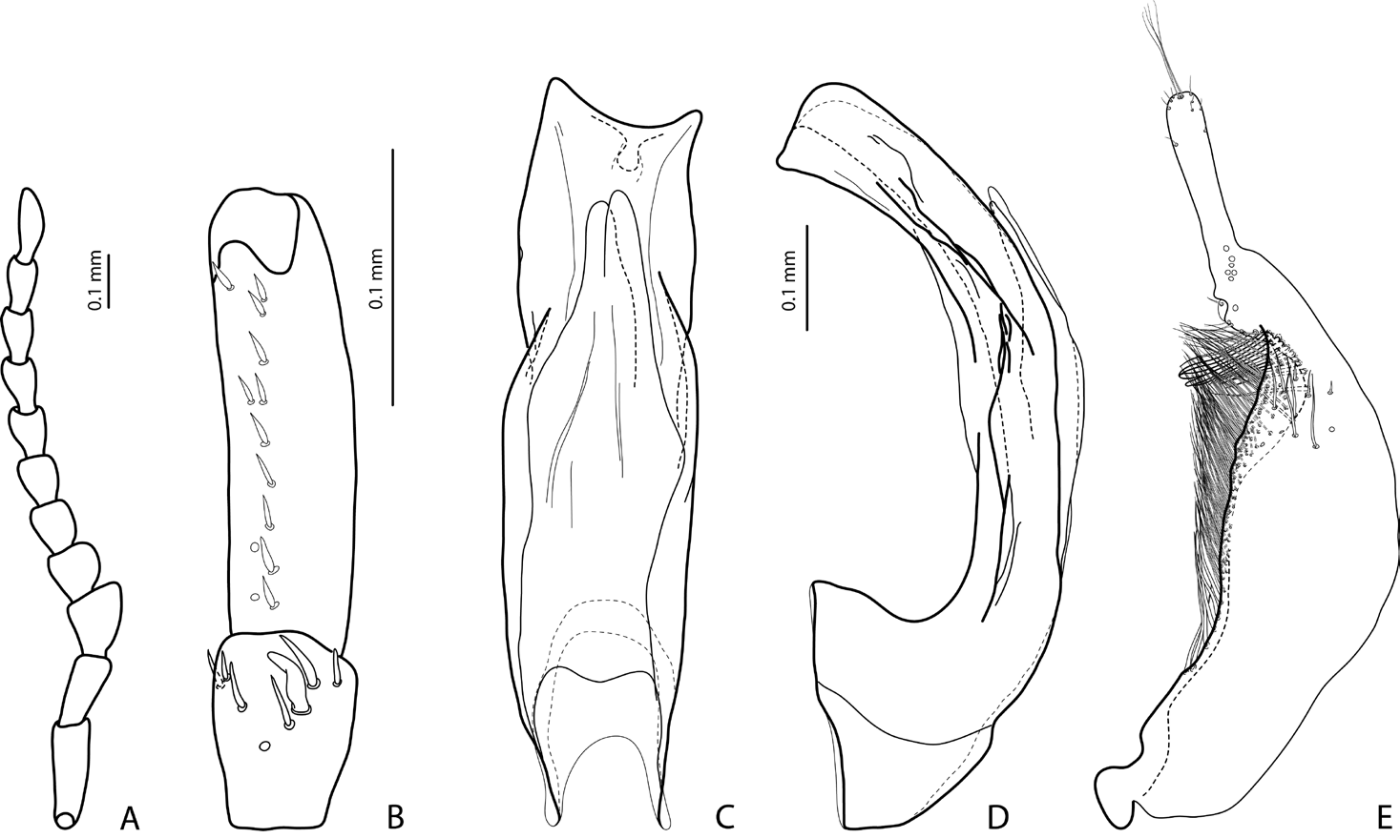
*Exocelina manokwariensis nokensis* ssp. n. **A** male antenna **B** male protarsomeres 4–5 in ventral view **C** median lobe in ventral view **D** median lobe in lateral view **E** paramere in external view.

*Male*: Protarsomere 5 ventrally with anterior row of 9 and posterior row 4 short setae (Fig. 8B). Abdominal ventrite 6 with 3–6 lateral striae on each side. Median lobe slightly larger and somehow more slender. Its apex with relatively short tip and truncate margin almost straight in lateral view and asymmetric in ventral view (Fig. 8C, D). Subdistal part of paramere with less numerous, thick flattened setae (Fig. 8E).

*Female*: Antennae simple, abdominal ventrite 6 broadly rounded apically, without striae.

#### Comparation with the other subspecies and co-inhabiting species.

Although widely separated geographically, *Exocelina manokwariensis nokensis* ssp. n. is very similar to the nominative subspecies, from which can be separated by the larger and somehow more slender median lobe, less numerous flattened setae on the paramere, and its finer dorsal microreticulation. This subspecies was collected together with two other species of the *Exocelina ekari*-group: *Exocelina evelyncheesmanae* Shaverdo, Hendrich & Balke, 2012 and *Exocelina waigeoensis* Shaverdo, Hendrich & Balke, 2012; see descriptions and illustrations in Shaverdo et al. 2012. From the former (both males and females), it can be easily separated by its smaller size and distinctly modified male antennae (TL-H: 3.75–4.1 mm, MW: 1.9–2.2 mm and slightly modified male antennae: antennomeres 3–7 very slightly enlarged, antennomere 3 slightly more triangular than other antennomeres, in *Exocelina evelyncheesmanae*). From the latter, males can be distinguished by their distinctly modified antennae and the shapes of the median lobes (slightly modified antennae and apex of the median lobe elongate in lateral view in *Exocelina waigeoensis*).

#### Distribution.

**Figure 9.**
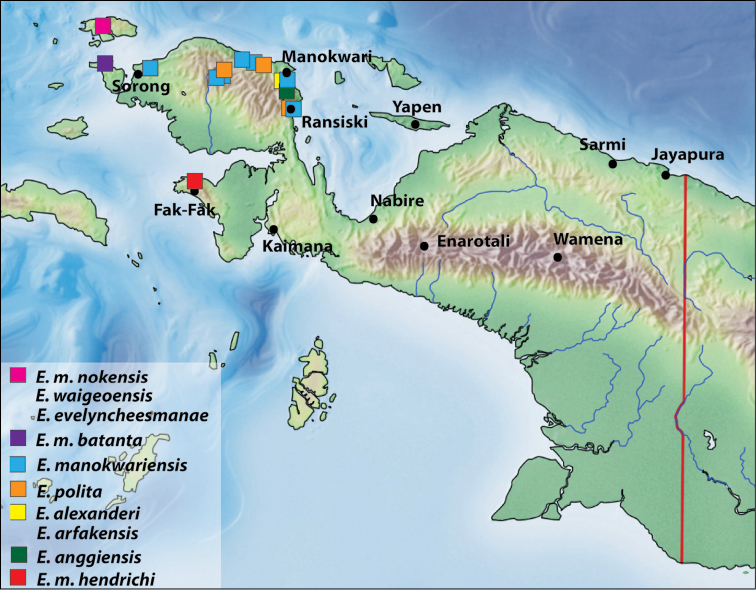
Map of the western part of New Guinea showing distribution of subspecies of *Exocelina manokwariensis* sp. n. and co-occurring species.

Indonesia: West Papua Province: Raja Ampat Regency, Waigeo Island (Fig. 9).

#### Etymology.

The species is named after Nok Mountain, the type locality of the species. The name is an adjective in the nominative singular.

#### Ecology.

The species was collected in two localities on Nok Mountain together with *Exocelina evelyncheesmanae* and *Exocelina waigeoensis* and in one locality together with *Exocelina evelyncheesmanae* in ration ca. 1:1.

### Key to subspecies of the *Exocelina manokwariensis* sp. n.

This key is a modified part of the key to species of the *Exocelina ekari*-group from Shaverdo et al. (2014). It is based mostly on male characters. In many cases, females cannot be assigned to species due to similarity of their external and internal structures (for female genitalia, see figs 17a and 17b in Shaverdo et al. (2005) and fig. 7C in Shaverdo et al. (2013)).

**Table:** 

27	Male antennomere 3 much larger than other antennomeres, triangular; beetle larger, TL-H: 3.8–4.8 mm, MW: 2.0–2.55 mm; male protarsomere 4 with anterolateral hook very small (smaller than more laterally situated large seta), thin, and slightly curved;paramere distinctly longer than median lobe, without notch on dorsal side, with dense, thin setae subdistally and sparse, thin setae and spines proximally	**28**
–	Male antennomeres 3 and 4 much larger than other antennomeres, triangular; beetle smaller, TL-H: 3.1–4.3 mm, MW: 1.65–2.3 mm; male protarsomere 4 with anterolateral hook thin or thick, slightly curved but larger than more laterally situated large seta; paramere equal or shorter than median lobe, with notch on dorsal side, setae of subdistal part thick and flattened, proximally setae dense and thinner, no spines	**29A**
29A	Beetle larger, TL-H: 3.7–4.3 mm, MW: 2.05–2.3 mm; dorsal punctation very fine to dense and coarse; apex of median lobe elongate in lateral view (figs 9–11 in Shaverdo et al. (2012)); if almost truncate, *Exocelina alexanderi*, antennomeres 3 and 4 larger, triangular but elongated due to external margin strongly expanded (figs 8A, D in Shaverdo et al. (2012))	**30**
–	Beetle smaller, TL-H: 3.1–3.85 mm, MW: 1.65–2.1 mm; dorsal punctation very fine; apex of median lobe almost truncate in lateral view (Figs 2D, 4D, 6D, 8D); male antennomeres 3 and 4 evidently smaller, more distinctly triangular, not elongated due to external margin only slightly expanded (Figs 2A, 4A, 6A, 8A)	***manokwariensis* sp. n. 32A**
32A	Apex of median lobe more strongly concave, with tip distinctly longer in lateral view (Fig. 6A, D)	***manokwariensis hendrichi* ssp. n.**
–	Apex of median lobe not concave or slightly concave, with tip shorter in lateral view (Figs 2A, 4A, 8A)	**32B**
32B	Apex of median lobe with truncate margin more strongly curved in lateral view and symmetrical in ventral view; subdistal part of paramere larger, with flattened setae less numerous and thinner (Fig. 4C–E)	***manokwariensis batanta* ssp. n.**
–	Apex of median lobe with truncate margin almost straight in lateral view and asymmetrical in ventral view; subdistal part of paramere smaller, with flattened setae more numerous and thicker	**32C**
32C	Median lobe longer, somehow more slender, subdistal part of paramere with less numerous flattened setae (Fig. 8C–E)	***manokwariensis nokensis* ssp. n.**
–	Median lobe shorter and more robust, subdistal part of paramere with more numerous flattened setae (Fig. 2C–E)	***manokwariensis manokwariensis* ssp. n.**

## Supplementary Material

XML Treatment for Exocelina manokwariensis

XML Treatment for Exocelina manokwariensis batanta

XML Treatment for Exocelina manokwariensis hendrichi

XML Treatment for Exocelina manokwariensis nokensis
